# Synovial tissue biopsy analysis: unlocking the hidden secrets to personalised medicine?

**DOI:** 10.1186/s13075-019-1871-5

**Published:** 2019-04-08

**Authors:** Helen Michelle McGettrick

**Affiliations:** Institute of Inflammation and Ageing, College of Medical and Dental Sciences, University of Birmingham, B15 2TT, Birmingham, UK

**Keywords:** Synovial biopsy, Rheumatoid arthritis, Synovial tissue analysis, Standardisation, Fibroblast, STB, RA

## Abstract

Rheumatoid arthritis is an immune-mediated inflammatory disease of the synovium. Yet we still lack robust tissue-specific (synovial) biomarkers that are able to guide clinical decisions and stratify patients according to their disease subgroup and predicted response to treatment. The EULAR Synovitis Study Group and the OMERACT synovial tissue biopsy (STB) Special Interests Group have undertaken a consensus exercise to identify factors that are important for the standardisation of STB handling and analytical procedures in two situations—clinical practice and translational research.

## Editorial

Rheumatoid arthritis (RA) is a heterogeneous disease in its clinical manifestations and bio-molecular pathology. The fact that not all patients are the same makes predicting disease onset, progression and severity, and response to treatment extremely challenging. Despite several attempts to identify blood-based biomarkers to guide clinical decision-making such as treatment choices, these have generally proven to be unreliable and inconclusive. Given the synovium is the site of disease, have we been and are we still looking in the wrong tissue? Detailed analysis of synovial tissue biopsies (STB) is likely to reveal urgently needed, robust biomarkers that will guide clinical decisions, and stratify patients according to their disease subgroup and predicted response to treatment. With this in mind, Najm and colleagues have undertaken a consensus exercise to identify factors that are important for the reliability and reproducibility of STB analysis across centres [1].

Since 1990, 1000’s of papers have been published describing phenotypic and functional abnormalities in synovial tissue from RA patients undergoing joint replacement surgery, and more recently those having STB at the earliest phases of disease. Thus, we have a wealth of knowledge on the synovial tissue, but are we closer to translating this discovery science into new biomarkers or drugs to improve diagnosis or prognosis (e.g. response to treatment, remission)? If we look at the story of one of my favourite cells, the tissue-resident synovial fibroblast (RASF): Early evidence demonstrated that RASF acquire a highly migratory, invasive phenotype [2, 3] and they appear to display tropism for damaged tissue, migrating to distant cell-free cartilage in vivo potentially ‘spreading’ disease [4]. Technological advances revealed epigenetic changes imprinted into RASF contribute to their aggressive phenotype (see review [5]) and that RASF themselves are heterogeneous, existing as at least 3 functionally distinct subpopulations in the synovium [6]. More recently, multi-cellular in vitro modelling demonstrated that synovial fibroblast communication with the blood vascular endothelium evolves as disease progresses to shape the inflammatory infiltrate, and revealed the presence of transitional functional phenotype in fibroblasts in the earliest phases of disease, even before clinical diagnosis [7]. The fibroblast journey has culminated in the identification of an anatomically distinct ‘pathogenic’ subpopulation of synovial fibroblasts in RA patients and in murine models of arthritis, which when injected in vivo induced a more prolonged and severe murine arthritis [8]. So what? Can we translate these findings into new biomarkers and/or therapies to influence clinical practice and patient outcomes? For further information on the use of STB in drug development see this review [9].

STB are well-tolerated by patients, with the majority willing to undergo repeat biopsy [10] allowing comparative pre and post intervention STB analysis. Three sides of our square are in place: clinical expertise, technology, and patient willingness. However, the fourth—standardisation in the handling, evaluation and interpretation of STB—requires considerable effort from the international rheumatology community to reach consensus. Najm et al. have now established a list of consensus points for standardised STB handling and analytical procedures in two situations—clinical practice and translational research [1]—and upon which a standardisation framework can be built. This task force used a modified Delphi analysis (structured anonymised questionnaire answered independently by a panel of experts) considering factors linked to biopsy sampling, processing, histological criteria, immunohistochemistry analysis, STB interpretation and pathologists report for both situation, and also RNA analysis for translational research [1]. For the full list of items Figure 2 in Najm et al., 2018 [1], but it is worth noting that the majority of items are identical for both clinical practice and translational research. Despite this, further agreements on the minimal area to biopsy; the quality of the STB (e.g. minimal number of vessels, or percentage of stroma); minimal thickness of STB sections; inclusion of CD15 as an indicator of infectious arthritis; or the use of vascularity as a parameter in clinical practice or translational research need to be achieved [1].

On the brink of establishing international standardisation guidelines for STB handling and evaluation in clinical practice and translational research, several additional factors now need to be deliberated by the community: the creation of a histology quality scoring system, and its subsequent validation to reveal the relationship between the synovial immunopathology and a patient’s response to therapy [11]. By way of example, three major synovial phenotypes have been described [12], with one (myeloid) associated with a good response to TNFi treatment [13]. Beyond this, we need novel STB biomarkers that define disease pathotype and outcome, and that correlate with blood-based biomarkers and imaging techniques to provide quicker, less invasive approaches for routine clinical use and outcome measures in clinical trials.
